# Angiogenesis, Lymphangiogenesis, and Inflammation in Chronic Obstructive Pulmonary Disease (COPD): Few Certainties and Many Outstanding Questions

**DOI:** 10.3390/cells11101720

**Published:** 2022-05-23

**Authors:** Remo Poto, Stefania Loffredo, Francesco Palestra, Gianni Marone, Vincenzo Patella, Gilda Varricchi

**Affiliations:** 1 Department of Translational Medical Sciences, University of Naples Federico II, 80131 Naples, Italy; remo.poto@gmail.com (R.P.); stefania.loffredo2@unina.it (S.L.); f.palestra97@gmail.com (F.P.); marone@unina.it (G.M.); 2 Center for Basic and Clinical Immunology Research (CISI), University of Naples Federico II, 80131 Naples, Italy; 3 World Allergy Organization (WAO), Center of Excellence, 80131 Naples, Italy; 4 Institute of Experimental Endocrinology and Oncology (IEOS), National Research Council, 80131 Naples, Italy; 5 Division of Allergy and Clinical Immunology, Department of Medicine, “Santa Maria della Speranza” Hospital, 84091 Battipaglia, Italy; patella@allergiasalerno3.it

**Keywords:** angiogenesis, angiopoietin, COPD, lymphangiogenesis, macrophage, vascular endothelial growth factor

## Abstract

Chronic obstructive pulmonary disease (COPD) is characterized by chronic inflammation, predominantly affecting the lung parenchyma and peripheral airways, that results in progressive and irreversible airflow obstruction. COPD development is promoted by persistent pulmonary inflammation in response to several stimuli (e.g., cigarette smoke, bacterial and viral infections, air pollution, etc.). Angiogenesis, the formation of new blood vessels, and lymphangiogenesis, the formation of new lymphatic vessels, are features of airway inflammation in COPD. There is compelling evidence that effector cells of inflammation (lung-resident macrophages and mast cells and infiltrating neutrophils, eosinophils, basophils, lymphocytes, etc.) are major sources of a vast array of angiogenic (e.g., vascular endothelial growth factor-A (VEGF-A), angiopoietins) and/or lymphangiogenic factors (VEGF-C, -D). Further, structural cells, including bronchial and alveolar epithelial cells, endothelial cells, fibroblasts/myofibroblasts, and airway smooth muscle cells, can contribute to inflammation and angiogenesis in COPD. Although there is evidence that alterations of angiogenesis and, to a lesser extent, lymphangiogenesis, are associated with COPD, there are still many unanswered questions.

## 1. Introduction

In 1628, William Harvey discovered that blood flows throughout the human body, being pumped by the heart through a single system of arteries and veins [1]. In 1661, Marcello Malpighi first identified the capillaries and, at the same time, Caspar Aselius discovered the lymphatic vessels [1]. Angiogenesis, a term coined by John Hunter in 1787, is the outgrowth and proliferation of capillaries from pre-existing blood vessels [2,3]. This process is distinct from other major forms of neovascularization, in which new blood vessels are formed from endothelial precursor cells (EPCs), also known as angioblasts [2,4].

EPCs share an origin with hematopoietic progenitors and assemble into a primitive vascular labyrinth of small capillaries in a process known as vasculogenesis [1]. The vascular plexus progressively expands by means of vessel sprouting and remodels into a highly organized vascular network of larger vessels ramifying into smaller ones. Nascent endothelial cell (EC) channels become covered by pericytes (PCs) and smooth muscle cells (SMCs), which provide strength and allow for the regulation of vessel perfusion; this neovascularization is known as arteriogenesis. The mammalian lymphatic system develops in parallel, but secondarily, to the blood vascular system through a process known as lymphangiogenesis [5]. Blood vessels arose early in evolution, whereas lymphatic vessels are present only in amphibians onwards [6].

After birth, angiogenesis contributes to organ growth, but during adult life, most blood vessels remain quiescent, and angiogenesis occurs physiologically only in the cycling ovary and in the placenta [5,7]. However, ECs retain their remarkable ability to rapidly divide in response to various stimuli, such as hypoxia and inflammatory mediators [7]. Angiogenesis and lymphangiogenesis can be reactivated during wound healing and repair and are tightly controlled by various factors. Their dysfunctionality contributes to different pathological conditions, including inflammatory disorders, infectious diseases, and cancer [3]. In several disorders, various biochemical, immunological, and physical stimuli can shift the balance between stimulators and inhibitors, resulting in an angiogenic or a lymphangiogenic switch. The best-known conditions in which these alterations are seen are malignant [8,9,10] and inflammatory disorders [11,12].

## 2. Proangiogenic and Antiangiogenic Factors

During the last decades, biochemical, genetic, and immunological studies have provided insights into the fundamental mechanisms and molecular players of angiogenesis [4] and lymphangiogenesis [13,14]. Vascular endothelial growth factor (VEGF), previously known as vascular permeability factor (VPF) [15], is the most specific growth factor for vascular endothelium [4]. VEGF is not a single protein but a family of several peptides [16], including VEGF-A, -B, -C, -D, and placental growth factor (PlGF) [17]. VEGF-A and -B are key regulators of blood vessel growth, and VEGF-C and -D primarily regulate lymphangiogenesis [18,19,20]. Some components of the VEGF family have additional splice variants that differ in their effects on angiogenesis. For example, human VEGF-A has at least six isoforms: 121, 145, 165, 183, 189, 204; VEGF-A_165_ is the most potent proangiogenic isoform [21]. PlGF, expressed in placenta and certain tumors, has two major isoforms, PlGF-1 (PlGF_131_) and PlGF-2 (PlGF_152_) [22].

**Figure 1:**
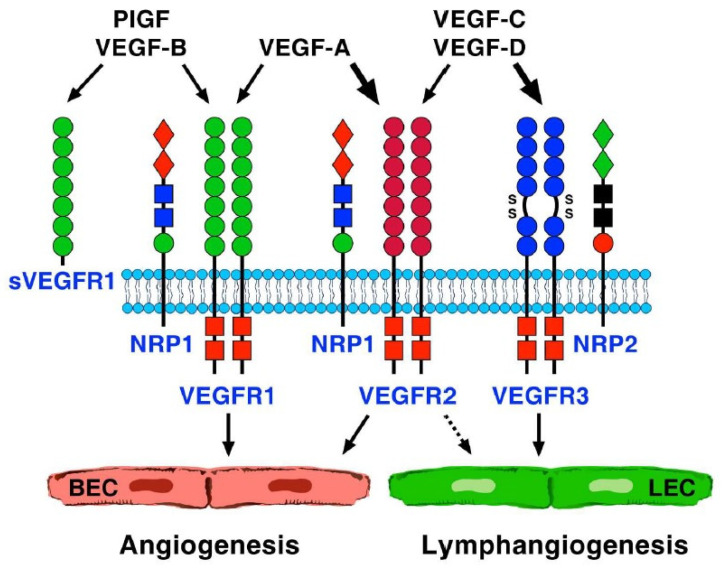
The family of vascular endothelial growth factors (VEGFs) and their receptors. This family of structurally related molecules includes VEGF-A, -B, -C, -D, and placental growth factor (PlGF). VEGF-A is the main mediator of angiogenesis. Several isoforms of VEGF-A activate tyrosine kinase receptors VEGFR1 and VEGFR2. VEGF-A signals mainly through VEGFR2, which is expressed at high levels by blood endothelial cells (BECs). VEGF-B and PlGF specifically activate VEGFR1: its role in angiogenesis, also expressed in BEC, is less clear. PlGF and VEGF-B also bind to soluble VEGFR1 (sVEGFR1). VEGF-C and -D activate VEGFR3 and VEGFR2. VEGFR3 is largely restricted to lymphatic endothelial cells (LECs). Besides the three tyrosine kinase receptors, there are co-receptors for VEGFs such as neuropilins (NRPs). NRP1 associates with VEGFR1 and VEGFR2 to bind VEGF-A, -B, and PlGF. NRP2 associates with VEGFR3 to bind VEGF-C and -D to regulate lymphangiogenesis.

VEGFs bind to three human members of the VEGF receptor (VEGFR) family, VEGFR1, 2, and 3, expressed by blood endothelial cells and/or lymphatic endothelial cells (LECs) [4,18,19,20]. VEGF functions are also modulated by an alternative mRNA variant of VEGFR1, soluble VEGFR1 (sVEGFR1). In addition to the VEGF receptors, two other molecules, Neuropilin 1 (NRP1) and -2 (NRP2) have been identified as co-receptors for VEGF [23]. NRP1 is mainly expressed by arterial ECs [24], whereas NRP2 is expressed by venous and lymphatic ECs [25]. NRP1 increases the affinity of VEGF-A_165_ for VEGFR2 and its phosphorylation, enhancing downstream signaling [26] (Figure 1).

**Figure 2:**
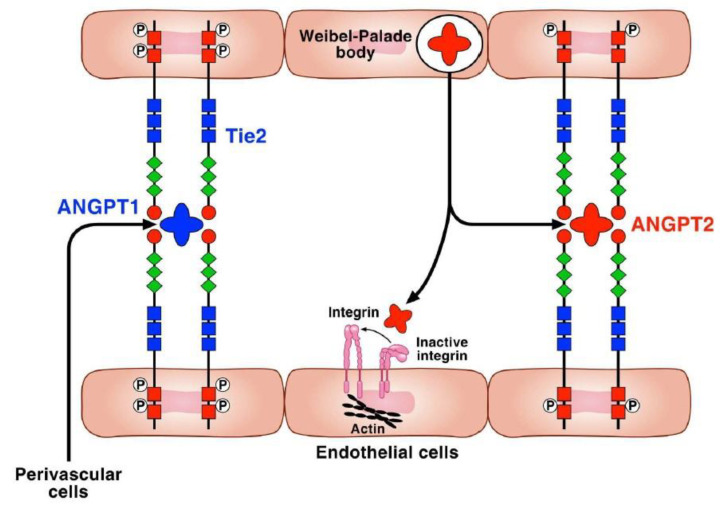
The angiopoietin/Tie receptor system. Angiopoietin 1 (ANGPT1), released by perivascular cells, is a Tie2 agonist that induces the formation of Tie2 clusters on endothelial cells (ECs) [39]. The phosphorylation of Tie2 leads to the activation of several downstream signaling pathways involved in vessel stability and endothelial barrier function. Thus, Tie2 activation by ANGPT1 promotes EC survival, migration, and proliferation, and inhibits vascular permeability [39,40]. By contrast, ANGPT2, released from EC Weibel–Palade bodies in response to various stimuli [41,42], activates Tie2 only weakly [43] and antagonizes ANGPT1 at the Tie2 receptor. Therefore, ANGPT2 increases vascular permeability and exerts proinflammatory effects [41]. ANGPT1 and/or ANGPT2 are also released from human macrophages [44,45], mast cells [9], and neutrophils [46]. ANGPT2 may also bind to and signal via integrin heterodimers [47,48]. The Tie1, homolog of Tie2, is considered an orphan receptor expressed by ECs.

Angiopoietins (ANGPTs) are members of another family of naturally occurring promoters of embryonic and postnatal neovascularization [27]. In humans, the ANGPT/Tie signaling pathway includes the Tie1 and Tie2 receptors and ANGPT1 and 2 [28]. Tie2 is a tyrosine kinase receptor highly expressed in ECs [29], but is also found in certain immune cells such as human basophils and lung mast cells (HLMCs) [30]. Tie1 is considered an orphan receptor without a known ligand (Figure 2). Angiopoietin-1 (ANGPT1), expressed by pericytes and other vascular supporting cells, promotes angiogenesis by establishing and maintaining vascular integrity and quiescence [31]. ANGPT1 binds to the Tie2 receptor on ECs and stabilizes nascent vessels by protecting the adult vasculature against plasma leakage induced by VEGF-A [31,32,33]. ANGPT2 is produced by ECs and acts in an autocrine manner as a partial Tie2 agonist (i.e., it quenches Tie2 signaling in the presence of ANGPT2 and weakly activates Tie2 in the presence of ANGPT1) [34]. Therefore, as an antagonist of constitutive ANGPT1/ANGPT2 signaling, ANGPT2 reduces vascular integrity [35,36]. Tie2 mRNA protein is most abundant in the lung, which is uniquely dependent on Tie2 signaling [32]. ANGPT2 increases plasma and alveolar fluid in acute lung injury and is a mediator of epithelial necrosis with an important role in hyperoxic lung injury and pulmonary edema [37]. ANGPT1 overexpression also induces lymphatic vessel enlargement, sprouting, and proliferation of LECs [38] (Figure 2).

Hepatocyte growth factor (HGF) is a potent inducer of tumor growth and the formation of metastasis [49]. HGF is secreted as an inactive precursor (pro-HGF), and proteolytic cleavage results in an active α- and β-chain heterodimer, which activates the tyrosine kinase receptor MET. HGF plays a major role in the modulation of angiogenesis and tumorigenesis [49]. Angiogenin is one of the most potent tumor-derived angiogenic factors [50,51]. It has been implicated as a mitogen for ECs, an immune modulator with suppressive effects on polymorphonuclear leukocytes, an activator of specific protease cascade, and an adhesion molecule [52]. Angiogenin is produced by macrophages, ECs, and peripheral blood lymphocytes [53].

Basic fibroblast growth factor (bFGF) belongs to a group of heparin-binding growth factors that stimulate EC proliferation and migration in vitro and angiogenesis in vivo [54]. bFGF plays a role in inflammatory conditions, wound healing [55], and pulmonary fibrosis [56]. There is evidence that angiogenin negatively regulates the expression of bFGF [54].

Several cytokines and chemokines are involved in angiogenesis [57]. CXCL8, secreted by human neutrophils [46,58], monocytes/macrophages [59], and mast cells [60], is a potent mediator of angiogenesis [61]. CXCL8 has several proangiogenic properties: it boosts EC proliferation [62] and induces EC chemotaxis and survival [61,63].

Several homologous proteins with similar or different biological profiles (IL-17B, IL-17C, IL-17D, IL-17E, IL-17F) are grouped in a cytokine family [64,65]. IL-17 promotes angiogenesis in humans by stimulating EC migration and regulating the production of various proangiogenic factors [66,67]. The promotion of angiogenesis by IL-17 may also result from enhancement of the action of bFGF, HGF, and VEGF-A [68].

Cysteinyl leukotrienes (cysLTs) are lipid mediators produced by human mast cells [69,70] and basophils [71] through the 5-lipoxygenase pathway [72]. Cys-LTs exert potent biological effects by activating the cys-LT receptors (cysLTRs). There is evidence that cysLTs can promote angiogenesis via the activation of cysLT_2_R [73,74,75]. Interestingly, a cysLT_2_R antagonist significantly reduced angiogenesis, suggesting that this receptor could be a possible target in the modulation of angiogenesis [75].

Different angiogenesis inhibitors have been identified. Thrombospondin-1 (TSP1), the first antiangiogenic factor identified in the 1990s, prevents VEGF-A-induced angiogenesis by directly binding to it and interfering with its binding to cell-surface heparan sulfates [76]. TSP1 is a potent inhibitor of EC migration and proliferation and an inducer of endothelial apoptosis [77]. Endostatin, a potent endogenous angiogenesis inhibitor [78], blocks endothelial growth and migration and promotes apoptosis. It antagonizes VEGF-A effects [79] at the VEGFR2 level [80]. VEGF-A mRNA splicing generates two protein families that differ by their carboxy-terminal six amino acids named VEGF-A_165a_ and VEGF-A_165b_ [81,82]. VEGF-A_165a_ is the canonical pro-angiogenic isoform, whereas VEGF-A_165b_ is the anti-angiogenic isoform. VEGF-A_165b_ binds to VEGFR2 but does not bind to NRP1. Therefore, VEGF-A_165b_ does not stimulate EC responses and inhibits several VEGF-A_165a_ -mediated EC processes [83,84]. VEGF-A_165b_ can be expressed and released by human neutrophils [46].

## 3. Chronic Obstructive Pulmonary Disease (COPD) and Inflammation

Chronic obstructive pulmonary (COPD) is a major global epidemic increasing worldwide as populations age [85,86]. COPD is the fourth-ranked cause of death worldwide, affecting approximately 10% of subjects older than 45 years [87]. There is compelling evidence that COPD is a complex and heterogeneous disorder with multiple endo-phenotypes and clinical presentations [88].

Inflammatory patterns in COPD have been referred to as inflammatory endotypes [89]. Neutrophilic inflammation is a hallmark of COPD and contributes to pivotal pathological features [88]. Eosinophilic inflammation can also be present as a stable endotype in a subgroup of COPD patients [90,91] and is associated with a favorable response to inhaled glucocorticoids (ICS) [92,93,94].

**Figure 3:**
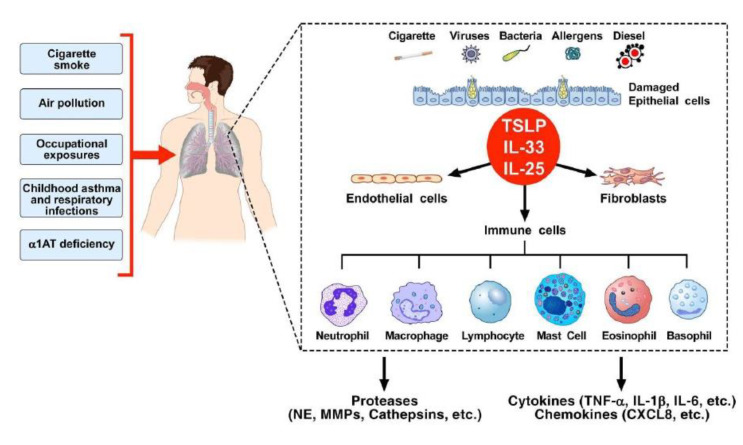
Schematic representation of the etiology and pathogenesis of COPD. Risk factors for COPD development include cigarette smoke, air pollution, occupational exposures, childhood asthma, respiratory infections, and alpha-1 anti-trypsin (α1AT) deficiency. Upon exposure to inhaled toxicants, lung structural cells, including epithelial cells and fibroblasts, as well as endothelial cells, are activated. Damaged bronchial epithelial cells release alarmins (TSLP, IL-33, IL-25) that activate several immune cells, endothelial cells, and fibroblasts. These cells produce inflammatory mediators to recruit other inflammatory cells, such as neutrophils, macrophages, and lymphocytes, to the site of exposure. This augments the expression of inflammatory mediators, such as cytokines (e.g., TNF-α, IL-6), chemokines [e.g., CCL2, CCL7, CXCL1, CXCL5, CXCL8], LTB_4_, and proteases (e.g., neutrophil elastase (NE), cathepsins, and matrix metalloproteinases (MMPs)). This cascade of events can lead to chronic pulmonary inflammation, airflow obstruction, and alveolar wall destruction (emphysema) in a susceptible individual.

Whatever the underlying disease mechanisms, COPD patients are characterized by chronic inflammation of the airways and lung parenchyma, occasionally associated with systemic inflammation [95]. Cigarette smoke is the primary cause of COPD and is responsible for approximately 70% of COPD cases [96]. Other risk factors for COPD are air pollution, occupational exposure, respiratory infections, childhood asthma, and α1 anti-trypsin (α1AT) deficiency [97] (Figure 3). Upon exposure to inhaled toxicants, bronchial epithelial cells (BECs) are activated and release preformed (i.e., alarmins: TSLP, IL-33, IL-25) and de novo synthesized cytokines (e.g., TNF-α, IL-1, IL-6, GM-CSF, and CXCL8). These mediators promote a cascade of signaling events leading to chronic pulmonary inflammation, airflow obstruction, and alveolar wall destruction in a susceptible individual.

Chronic airway inflammation in COPD is associated with the activation of tissue-resident (e.g., macrophages, mast cells) and -infiltrating immune cells (e.g., neutrophils, eosinophils, basophils, CD8^+^ T lymphocytes) in the lumen and wall of airways and parenchyma [88,98,99,100]. COPD development can also invoke pulmonary vascular remodeling. Pulmonary arteries show structural abnormalities even in mild COPD without arterial hypoxemia and in smokers with normal lung function [101]. There is also evidence that angiogenesis and, to a lesser extent, lymphangiogenesis, are dysregulated in COPD [102,103,104,105,106]. However, airway angiogenesis and lymphangiogenesis in COPD and emphysema have been surprisingly poorly studied considering the relevance of these conditions.

## 4. Epithelial-Derived Cytokines

Thymic stromal lymphopoietin (TSLP) is an epithelial-derived cytokine expressed in airway biopsies of COPD patients [107]. TSLP is an early player in triggering airway inflammation via the activation of several immune cells (e.g., DCs, ILCs, monocytes, macrophages, mast cells) [108,109,110]. TSLP immunoreactivity is greater in ASM samples from COPD patients compared to healthy subjects [111]. Intranasal exposure to cigarette smoke extract [112] in mice increases mRNA and protein expression of TSLP [113]. CSE induces the mRNA expression and TSLP release from human ASM cells [114]. We have recently demonstrated that TSLP induces the release of several angiogenic (i.e., VEGF-A, ANGPT2) and lymphangiogenic factors (i.e., VEGF-C) from HLMs [20].

IL-33 concentrations are increased in serum, sputum, and bronchial biopsy samples of COPD patients [115,116,117,118,119]. Serum IL-33 levels were associated with COPD exacerbations [120]. Moreover, in a preclinical mouse model of COPD, exacerbations induced by CSE and viral infections promoted intracellular accumulation of IL-33 [116]. Finally, combustion-generated ultrafine particulate matter induced IL-33 release from peripheral blood mononuclear cells (PBMCs) [121]. There is ample evidence that IL-33 can induce angiogenesis in different experimental models [122,123].

In a preclinical mouse model, IL-25 (also known as IL-17E) activated innate lymphoid cells (ILC2s)-2 and caused pulmonary fibrosis [124]. Elevated concentrations of TSLP, IL-25, and IL-33 have been found in induced sputum of patients with COPD compared to controls [125]. To distinguish between asthma and COPD, which sometimes might be difficult in clinical practice, several biomarkers have been analyzed in the two groups of patients [126]. The authors found that asthma patients were characterized by higher levels of FeNO and peripheral blood eosinophils. It has been reported that IL-25 has the potential to promote angiogenesis [127].

## 5. Inflammatory Cells, Angiogenesis, and Lymphangiogenesis

The inflammation seen in the lungs of COPD patients involves both innate (macrophages, neutrophils, mast cells, eosinophils, basophils, natural killer cells (NK cells), γδ T cells, ILCs, and dendritic cells (DCs)) and adaptive immunity (B and T lymphocytes) [85]. There is also evidence that structural cells, including BECs and alveolar epithelial cells, ECs, fibroblasts, and myofibroblasts, can contribute to inflammatory mechanisms and angiogenesis in COPD.

### 5.1. Macrophages

Macrophages are the predominant immune cells in the human lung parenchyma and are the first line of defense against pollutants and microbial pathogens [20,45]. These cells play a fundamental role in orchestrating chronic inflammation in COPD (Figure 1). Macrophage density is markedly increased (up to 10-fold) in the airways, lung parenchyma, bronchoalveolar lavage (BAL) fluid, and sputum of COPD patients. Macrophages can be activated by CSE to release inflammatory mediators, including cytokines (e.g., TNF-α), chemotactic factors [CXCL1, CXCL8, CCL2, LTB_4_], and reactive oxygen species (ROS). Alveolar macrophages also secrete elastolytic enzymes, including matrix metalloproteinases (MMPs) -2, -9, and -12, cathepsins, and elastase [128]. MMP-9 is the predominant elastolytic enzyme secreted by alveolar macrophages from patients with COPD.

Compelling evidence indicates that human macrophages are highly heterogeneous [129,130,131,132,133]. In the human lung, several subsets of macrophages have been identified [130,131,132,134], and it has been suggested that M1-like macrophages predominate in COPD patients [135]. However, further studies using single-cell RNA sequencing are needed to characterize the macrophage subpopulations in COPD patients. Recent evidence indicates that rhinovirus impairs the innate immune response to different bacteria in alveolar macrophages from patients with COPD [136]. Human rhinovirus also induced the release of cytokines (e.g., IL-6, TNF-α, IL-10, CXCL8) from macrophages.

Primary human macrophages (HLMs) purified from human lung parenchyma express angiogenic (VEGF-A and -B) and lymphangiogenic factors (VEGF-C and -D) [45,137]. Secretory phospholipase A_2_ (sPLA_2_), an enzyme expressed in the airways of patients with lung diseases including COPD [138], enhances the expression and release of VEGF-A and -C from HLMs. HLMs activated by lipopolysaccharide (LPS) release VEGF-A, ANGPT1, ANGPT2, and VEGF-C [45]. We have also found that sPLA_2_ induces the release of VEGF-A, ANGPT1, ANGPT2, and CXCL8 from HLMs [139].

Recently, we have found that two TSLP isoforms (long (lfTSLP) and short (sfTSLP)) and a TSLP receptor (TSLPR) are expressed in HLMs [20]. TSLP, contained in HLMs, was released in response to LPS. These results prompted us to investigate whether HLMs could be a target of TSLP. We found that TSLP induced the release of angiogenic (VEGF-A and ANGPT2) and lymphangiogenic factors (VEGF-C) from HLMs [20]. These results highlight a novel immunological network involving epithelial-derived TSLP, TSLPR, and the release of angiogenic and lymphangiogenic factors from HLMs.

### 5.2. Mast Cells

Mast cells are prominent immune cells in human lung parenchyma [12,69,132] and play a pivotal role in coordinating lung inflammation [140]. Mast cell density is increased in bronchial biopsies from COPD patients compared to healthy controls [99]. Activated rodent mast cells release VEGF-A and FGF-2 [141], and mast cell supernatants induce an angiogenic response in the chorioallantoic membrane [142,143]. HLMCs constitutively express other VEGFs in addition to VEGF-A, namely the angiogenic VEGF-B and the lymphangiogenic VEGF-C and -D [144]. These VEGFs are often present as preformed mediators in mast cells [9]. PGE_2_ and adenosine, two important proinflammatory mediators, induced the expression of VEGF-A, -C, and -D in HLMCs [144]. These findings indicate that HLMCs have an intrinsic capacity to produce several VEGFs, suggesting that these cells might regulate both angiogenesis and lymphangiogenesis. HLMCs are not only a source of VEGFs in the airways, but also a target for these angiogenic factors. Indeed, HLMCs express VEGFR1 and 2, two major receptors for VEGFs. Different VEGFs (VEGF-A, -B, -C, -D, and PlGF-1) exert chemotactic effects on HLMCs by engaging both receptors. Recently, we found that several bacterial superantigens can induce the release of angiogenic (VEGF-A) and lymphangiogenic (VEGF-C) factors from HLMCs. Interestingly, the epithelium-derived cytokine IL-33 potentiated the release of proinflammatory (i.e., histamine), angiogenic, and lymphangiogenic factors from HLMCs [145]. These results suggest that IL-33 might enhance the inflammatory angiogenic and lymphangiogenic activators of HLMCs in pulmonary disorders.

### 5.3. Neutrophils

Increased numbers of activated neutrophils are found in the sputum and BAL fluid of COPD patients and correlate with disease severity, although few neutrophils are found in the bronchial wall and lung parenchyma [88]. Smoking stimulates the production and release of neutrophils from bone marrow and survival in the respiratory tract, possibly mediated by GM-CSF and G-CSF secreted from lung macrophages. Neutrophils’ recruitment to the lung parenchyma involves initial adhesion to activated ECs through E-selectin, which is overexpressed on ECs in the airways of COPD patients. Neutrophils migrate into the respiratory tract under various chemotactic factors such as LTB_4_, CXCL1, CXCL5, and CXCL8 [146]. These chemotactic mediators can be derived from alveolar macrophages, mast cells, T cells, and epithelial cells [146]. Neutrophils themselves might be a major source of CXCL8 [58]. Neutrophils from COPD patients are activated and have increased concentrations of myeloperoxidase [142,147]. Activated neutrophils secrete neutrophil elastase (NE), cathepsin G (CG), and proteinase 3 (PR3), as well as MMP-8 and MMP-9, which contribute to alveolar destruction. NE, CG, and PR3 are potent promoters of mucus secretion from submucosal glands and goblet cells [148]. During COPD exacerbations, there is a marked increase of neutrophils in the airways, resulting in the increased production of neutrophil chemotactic factors (e.g., LTB_4_ and CXCL8) [149].

Activated human neutrophils release neutrophil extracellular traps (NETs) [150,151]. Increased components of NETs have been found in the sputum of both stable and exacerbating COPD patients, alongside an increased proportion of NET-producing neutrophils [152,153]. The abundance of NETs in sputum correlates with the severity of airflow limitation [152,154], loss of microbiota diversity [154], and overall severity of COPD [154]. Despite these observations, neutrophils isolated from the blood of patients with COPD exacerbations have an apparently reduced ability to form NETs compared to stable patients and healthy controls, despite the increased plasma levels of cell-free DNA [155]. Finally, it should be noted that NETs can directly and indirectly promote angiogenesis [8].

Human neutrophils constitutively express and contain several proangiogenic factors (VEGF-A_165_, VEGF-B, ANGPT1, CXCL8, and HGF) [46,156]. Human neutrophils, similarly to other circulating immune cells (e.g., basophils) [157], do not express lymphangiogenic factors (VEGF-C and -D). sPLA_2_ selectively induces the release of proangiogenic factors from human neutrophils [46]. Of note, sPLA_2_-activated neutrophils also express the antiangiogenic isoform VEGF_165b_ [46]. The relevance of the latter observation in the context of COPD remains to be defined. More recently, we found that LPS-activated neutrophils release VEGF-A, which stimulates angiogenesis through the formation of tubules in vitro [58].

### 5.4. Eosinophils

Eosinophils have been identified in different anatomical compartments of COPD-affected lungs and increased in severe patients [100]. However, the role of eosinophils and their mediators in COPD is still uncertain. Increased eosinophil numbers have been described in the airways and BAL fluid of patients with stable COPD, whereas others have not found increased numbers in airway biopsies, BAL fluid, or induced sputum [158]. The presence of eosinophils in COPD patients seems to predict a more favorable therapeutic response to bronchodilators and ICS [94] and might indicate coexisting asthma or asthma-COPD overlap syndrome (ACOS) [159,160,161]. Up to 15% of COPD patients appear to have clinical features of asthma [142]. The mechanism for increased eosinophil counts in some patients with COPD is debated [162]. It has been suggested that damaged BECs release IL-33, which can induce the release of IL-5 from ILC2s [163]. IL-33 expression is increased in basal epithelial progenitor cells in COPD patients and is associated with increased levels of IL-13 and the mucin gene 5AC [119]. IL-33 is expressed in the lungs of COPD patients [164], and levels of IL-33 and its receptors ST2 are increased in the serum of these patients. Moreover, circulating IL-33 levels in COPD patients are correlated to peripheral blood eosinophils [118]. Finally, the exposure of PBMCs from COPD patients to combustion-generated ultrafine particles obtained from fuel induced the release of IL-33 [121].

The potential role of eosinophils and their powerful mediators in the pathophysiology of certain COPD endotypes has generated some enthusiasm in treating this heterogeneous disorder with monoclonal antibodies (mAbs) targeting IL-5 (i.e., mepolizumab) or IL-5Rα (i.e., benralizumab). In COPD patients with eosinophilic phenotype, mepolizumab decreased the annual rate of exacerbations compared to a placebo group [165]. Benralizumab was not associated with a lower annualized rate of COPD exacerbations than placebo among patients with blood eosinophils counts ≥ 220 per mm^3^ [166].

Human eosinophils synthesize and store in their granules several proangiogenic mediators such as VEGF-A, FGF-2, TNF-α, GM-CSF, nerve growth factor (NGF), and CXCL8 [167]. In addition, these cells promote EC proliferation in vitro and induce vessel formation in aortic rings and in the chick CAM assays [168].

### 5.5. Basophils

Although human basophils account for 0.5–1% of all leukocytes in peripheral blood, these cells play critical roles in clearing pathogens [169,170,171], initiating allergic disorders [71,172], and COPD [100]. Basophil density is increased in the lung tissue of COPD patients compared to smoking controls [100]. A significant correlation was found between basophils and eosinophils in the lungs of COPD patients. Activated human basophils express several forms of VEGF-A (121, 165, 189), and their secretory granules contain VEGF-A [157]. The activation of human basophils induces the release of VEGF-A [157] and ANGPT1 [30]. Human basophils also express HGF [173]. VEGF-A has a chemotactic effect on basophils through the activation of VEGFR2. These cells do not express VEGF-C and -D and presumably play a role in angiogenesis, but not in lymphangiogenesis [157].

### 5.6. Lymphocytes

CD8^+^ and, to a lesser extent, CD4^+^ T cells, are increased in the lung parenchyma, bronchi, and bronchioles of COPD patients compared to asymptomatic smokers [174,175]. There is evidence that CD8^+^ T lymphocytes are both increased in number and have increased functional activity in COPD [175]. CXCR3 is highly expressed on effector T cells following activation by ligands such as CXCL10. CD8^+^ T lymphocytes themselves produce CXCL10, thus recruiting more CXCR3^+^ T cells to the lung, where they exert inflammatory and destructive effects. The overexpression of CXCR3 and its ligand CXCL10 by BECs could contribute to the accumulation of CD8^+^ and CD4^+^ T cells, which express CXCR3 [176].

ILCs are critical players in mucosal immunity. Group 1 ILCs (ILC1), group 2 (ILC2), and group 3 (ILC3) are a population of tissue-resident lymphocytes with pleiotropic roles in mucosal inflammation, including defense against pathogens, the maintenance of epithelial barrier homeostasis, the containment of microbiota, and tissue repair [177]. ILCs play an important role in the regulation of lung immunity and might be activated through danger signals and cell damage [178]. All three groups of ILCs have been identified in the human lung [179]. In COPD patients, there is an increase in the number of ILC3s, which secrete IL-17 and IL-22, and these cells might play a role in driving neutrophilic inflammation. Exposure to cigarette smoke inhibits ILC2 function, and this is associated with an exaggerated anti-viral response [116]. Moreover, exposure to cigarette smoke and viral infections induced the emergence of the ILC1 population in mice [180]. The same authors found that the frequency of circulating ILC1 was higher in COPD patients compared to healthy controls. Conversely, the frequency of ILC2 cells was lower in COPD patients compared to healthy smokers. A similar increase in ILC1 frequency has been reported in the lungs of COPD patients [181].

A distinct cluster of CD4^+^ T helper 17 (Th17) cells are characterized by the expression of the master transcription factor RORγt [65]. CD4^+^ T_H_17 cells, which secrete IL-17A and IL-22, are increased in the airways of COPD patients and might play a role in orchestrating neutrophilic inflammation [182,183]. Th17 cells produce the IL-17 family of structurally related cytokines, IL-17A through IL-17F. IL-17A, commonly known as IL-17, is the prototypical member of this family. It was reported that Th17 cells release IL-1β and IL-17 and exert lymphangiogenic effects [184]. IL-17A promotes angiogenesis in preclinical [66] and clinical models of vascular remodeling [185]. IL-17E (IL-25), a little unusual among the IL-17 family, is produced by bronchial epithelial cells [186] and tuft cells [187] and in respiratory viral infections [188].

B lymphocytes are also increased in the lungs of COPD patients, particularly in those with severe disease [189]. B cells can be organized into lymphoid follicles located in peripheral airways and lung parenchyma [190]. The expression of B-cell activating factor, an important regulator of B-cell function and hyperplasia, is increased in the lymphoid follicles of patients with COPD [191,192]. Recent evidence indicates that a subset of regulatory B cells (Bregs) with high levels of the surface markers CD24 and CD38, previously shown to exert immunosuppressive functions, is decreased in the peripheral blood of COPD patients [193].

### 5.7. Dendritic Cells

Dendritic cells (DCs) are an important link between innate and adaptive immunity [194]. The airways and lungs contain a rich network of DCs localized near the surface, so that they are ideally located to signal the entry of inhaled foreign substances [195,196]. Epithelium-derived cytokines (TSLP, IL-33, IL-25) are important modulators of DC functions [197,198,199]. DCs can activate a variety of other inflammatory and immune cells, including macrophages, neutrophils, and T and B lymphocytes, and therefore DCs might play an important role in the pulmonary response to cigarette smoke and other inhaled toxic chemicals [200]. DCs are activated in the lungs of COPD patients [201] and correlate to disease severity [202]. The numbers of DCs are increased in the lungs of COPD patients, and cigarette smoke increases their survival in vitro [203]. Human DCs can produce biologically active VEGF-A [194]. DCs activated by different bacteria release VEGF-A, which induces neutrophil recruitment to the site of inflammation [204].

### 5.8. NK Cells

NK cells, as innate immune cells, contribute to the first line of defense mechanisms for the human body against viral and bacterial infections and tumors [205]. NK cells have been implicated in maintaining immune homeostasis in the lung and in the pathogenesis of COPD [206,207]. However, the specific mechanisms of involvement of NK cells in COPD are still rather elusive [208]. NK cells make up 5–15% of the circulating lymphocytes. These cells are subdivided into two main subpopulations, CD56^bright^ CD16^−^ and CD56^dim^ CD16^+^. CD56^bright^ CD16^−^ NK cells, accounting for about 10% of peripheral blood NK population, mainly produce several cytokines (i.e., IFN-γ, IL-10, TNF-α, GM-CSF). CD56^dim^ CD16^+^ NK cells, the predominant (approximately 90%) peripheral blood NK cells, are highly cytotoxic by producing perforin and granzymes and inducing antibody-dependent cytotoxicity [205]. In humans, NK cells represent 5-20% of the CD45^+^ lung lymphocytes [209]. Approximately 80% of lung NK cells show the CD56^dim^ CD16^+^ phenotype, whereas the remaining 20% are CD56^bright^ CD16^−^ and CD56^dim^ CD16^−^ [207]. There is evidence that the low cytotoxic CD56^bright^ CD16^−^ phenotype exerts pro-angiogenic activity [210].

Several studies examining the frequency and activation status of NK cells in peripheral blood and induced sputum in COPD patients have provided contrasting results [208]. Thus, further studies are needed to elucidate the mechanisms of NK cells in the pathogenesis, endotypes, and exacerbations of COPD.

## 6. Structural Cells and Angiogenesis

### Epithelial Cells

The bronchial epithelium constitutes a key component of the innate immune system, providing a physical and immune-modulatory barrier that is a first line of defense against environmental agents. Epithelial cells are activated by cigarette smoke and other inhaled irritants (i.e., biomass fuel smoke) to produce a plethora of inflammatory mediators (e.g., TNF-α, IL-1β, IL-6, GM-CSF, and CXCL8) [211]. There is compelling evidence that viral and bacterial products [212,213,214,215], smoke extracts [113,114], diesel exhaust [216], and cytokines [217] can induce the rapid release of epithelial-derived cytokines (TSLP, IL-33, and IL-25), also known as alarmins. These upstream cytokines can activate several immune (e.g., DCs, ILCs, macrophages, mast cells, neutrophils, eosinophils) and structural [e.g., fibroblasts/myofibroblasts, ASM cells, goblet cells, and ECs] cells [108,123]. Thus, epithelial-derived cytokines might play an upstream role in airway remodeling in COPD. In particular, TSLP expression in bronchial biopsies was increased in COPD patients compared to healthy ex-smokers and smokers [107]. Moreover, most COPD exacerbations are associated with viral infections, and rhinoviral infection induced the overexpression of TSLP [218].

VEGFs appear to be necessary to maintain alveolar cell integrity, and their blockade in rats induces apoptosis of alveolar cells and an emphysema-like pathology [219]. A reduction in peripheral lung VEGF concentrations is found in smokers and COPD patients, but levels of HGF, another growth factor, are increased in smokers and therefore might protect against the effect of reduced VEGF levels on alveolar integrity. In COPD patients, both VEGF and HGF levels are reduced, which might contribute to the development of emphysema [220].

The airway epithelium in COPD patients often shows squamous metaplasia, resulting from increased proliferation of basal BECs. Epithelial growth factor receptors (EGFRs) show an increased expression in the BECs of COPD patients and might contribute to basal cell proliferation, resulting in squamous metaplasia and an increased risk of bronchial carcinoma [221]. Goblet cell hyperplasia, a typical feature of COPD, is a response to chronic airway insult due to cigarette smoke and other pollutants. EGFRs play an important role in mucus hyperplasia and secretion and can be activated by neutrophilic inflammation through NE secretion, which releases TGF-α [222] (Figure 2). Oxidant stress can also activate EGFRs and induce mucus hypersecretion [223].

Human BECs constitutively express and release significant amounts of VEGF-A in cell culture media at concentrations capable of stimulating EC growth. Hypoxia and TGF-β1 stimulated VEGF-A production in these cells [224]. Canine vascular smooth muscle cells (VSMCs) express VEGFR1, 2, and NRP1 at mRNA and protein levels and respond to VEGF-A in vitro [225]. These findings have been extended by showing that ASM cells also express several splice variants of VEGF-A (121, 165, 189, 206) and constitutively secrete VEGF-A protein [226]. Certain cytokines (e.g., IL-1β, TGF-β) increase VEGF-A production by human VSMCs [227]. VEGF stimulation enhanced the production of MMPs by human VSMCs [228], and VEGF-A can induce fibronectin secretion by human ASM cells. These findings suggest that lung structural cells can contribute to angiogenesis through the local release of angiogenic factors.

The endothelium has long been known to be dysfunctional in COPD [229]. Endothelial dysfunction is associated with COPD severity and is related to FEV_1_ and the percentage of emphysema on CT scans [230,231]. MicroRNAs (miR) are small non-coding ribonucleic acids (RNAs) that regulate gene expression [232]. miR expression differs between COPD patients and healthy controls [233,234]. A recent study identified three miR upregulated in COPD pulmonary endothelial cells: miR-181b-3p, -429, and -23c [234]. These miRs impair angiogenesis (tube formation and sprouting of endothelial cells). miR-driven changes in the pulmonary endothelium might represent a novel mechanism driving COPD through alterations in angiogenesis.

## 7. Angiogenesis and Lymphangiogenesis in Experimental Models of Chronic Airway Inflammation

The role of angiogenesis and lymphangiogenesis has been evaluated in different experimental models of COPD. A plethora of stimuli, including cigarette smoke [235], hypoxia [236], and cytokines (e.g., IL-1β and TGF-β) [227] increase VEGF-A production. Perfusion of isolated lungs under hypoxic conditions increased tissue VEGF-A and VEGFR1 and 2 mRNAs [237]. VEGF-A and VEGFR2 were similarly overexpressed in chronically hypoxic rats, suggesting that both acute and chronic hypoxia increase the lung tissue expression of VEGF-A and its receptors. The same investigators reported that the pharmacologic blockade of VEGFR boosted the expression of oxidative stress, alveolar cell apoptosis, and alveolar enlargement [238]. VEGF seems to protect ECs against apoptosis in models of rapidly growing vessels during fetal development or tumor angiogenesis [239].

Cigarette smoke is a complex mixture containing a myriad of oxidant molecules [240] and can increase oxidative stress. CSE down-regulates VEGF expression by epithelial cells, causes EC apoptosis, and shortens the VEGF-dependent survival of cultured ECs [238]. Chronic cigarette exposure or administration of a VEGFR antagonist caused alveolar cell apoptosis and airspace enlargement [238,241]. VEGF-B, a selective agonist of VEGFR1, is expressed in the lung [242] and can stimulate angiogenesis in the pulmonary circulation through the interaction with VEGFR1 [243]. The role of VEGF-B in experimental COPD models and in patients with this disorder is largely unknown.

Lymphatic vessel hyperplasia plays a role in chronic airway inflammation. In mouse models of chronic respiratory tract infection with *Mycoplasma pulmonis,* lymphangiogenesis began slowly in airway inflammation, but after a few weeks, it overtook the remodeling and proliferation of blood vessels and persisted after the resolution of inflammation [244]. Lymphangiogenesis in inflamed airways is mediated by VEGF-C and -D, mainly derived from airway-immune cells (e.g., macrophages and mast cells), [12,20,45,58,137,144,145] through VEGFR3 signaling in lymphatic ECs. Further studies are needed to clarify the factors modulating the growth and regression of lymphatic vessels in chronic airway inflammation.

## 8. Angiogenesis and Lymphangiogenesis in COPD

Initial studies in bronchial biopsies of COPD patients did not find any significant increase in vessel number [245,246]. A subsequent immunohistochemical study on bronchial biopsies from patients with moderate COPD (GOLD 2) showed an increase in the number of vessels and the vascular area compared to controls. The increase in bronchial vascularity was associated with higher cellular expression of VEGF-A [102]. The immunohistochemical expression of VEGF-A was also greater in pulmonary arteries of smokers with normal lung function and patients with moderate COPD than in non-smokers [106]. VEGF levels in serum and induced sputum were higher in COPD patients than controls [103,104,247]. An interesting case-control study revealed that genetic polymorphisms of HIF-1α and VEGF are associated with the progression of COPD [248].

Kranenburg and colleagues [105] found that COPD was associated with increased VEGF expression in bronchial, bronchiolar, and alveolar epithelium, lung macrophages, ASM cells, and VSMCs. VEGFR1 and 2 were also higher in COPD patients. The authors found an inverse correlation between VEGF and FEV_1_ and suggested that increased VEGF expression was an attempt to repair lung damage in COPD. COPD patients with acute exacerbations may have a transient increase in circulating concentrations of VEGF and C-reactive protein (CRP) and a higher neutrophil count than stable COPD and healthy controls [249].

The significance of the VEGF/VEGFR system in COPD and emphysema appears to differ. VEGF and its receptor VEGFR2 were decreased in lung extracts of emphysematous lungs [250]. Santos et al. examined surgical specimens from non-smokers, smokers with normal lung function, patients with moderate COPD, and patients with emphysema [106]. Although COPD patients showed an overexpression of VEGF, in patients with severe emphysema, the expression of VEGF-A in pulmonary arteries was low despite intense vascular remodeling. The authors suggested that VEGF-A expression varies with the severity of COPD and might be involved in pulmonary vascular remodeling at the early stages of the disease. VEGF expression in alveolar macrophages was downregulated in patients with emphysema compared to smokers without emphysema [251]. VEGF is a growth factor that maintains alveolar homeostasis, so the decrease in VEGF expression in emphysema might play a pathogenic role.

There is little information on factors besides VEGFs that could affect angiogenesis in COPD. It has been reported that the serum and BAL fluid levels of PlGF increase in COPD patients and are inversely correlated with FEV_1_ [252]. Immunochemistry studies found that the expression of bFGF in the airways of COPD patients was greater than in asymptomatic smokers [253].

Collectively, these findings support the hypothesis that angiogenesis is a prominent feature of airway inflammation in COPD: increased vascularity and enhanced bronchial expression of angiogenic factors (mostly VEGF-A) are associated with COPD development. VEGF-A overexpression appears inversely correlated to the disease severity.

COPD is frequently associated with other concomitant systemic disorders [85,254]. For example, limb skeletal muscle dysfunction affects the morbi-mortality of these patients [255]. Capillary remodeling in response to exercise training is linked to angiogenesis [256]. The skeletal muscle angiogenic process (i.e., capillary creation and maturation) of COPD patients in response to exercise training is impaired compared to controls [257]. Moreover, women with COPD from biomass smoke have reduced serum levels of biomarkers of angiogenesis and tumor progression (e.g., FGF-2, HGF, sVEGFR2, sHER2/neu, sTIE-2) compared to women with COPD from smoking [258].

## 9. Therapeutic Opportunities

Given the potential role of angiogenesis and lymphangiogenesis in COPD, further studies are needed to investigate the role of angiogenic and lymphangiogenic inhibitors as a therapeutic approach for COPD treatment. In a mouse model of COPD induced by LPS injection and cigarette smoke inhalation, sunitinib, a specific tyrosine kinase inhibitor, has been shown to downregulate the expression of VEGF, VEGFR1, and VEGFR2. In addition, it also downregulates the phosphorylation of VEGFR1/R2 [259]. Moreover, anti-angiogenic nanotherapy has been shown to inhibit airway remodeling in a mouse model of asthma [260]. There is increasing evidence that cysLTs, major lipid mediators produced by human mast cells [69,70] and basophils [71], can promote angiogenesis [73,74,75] via the activation of cysLT_2_R. A cysLT_2_R antagonist has been shown to inhibit angiogenesis [73,74,75]. This class of compounds should be investigated to assess their effects in preclinical models of COPD. High-dose ICS therapy (2000 mcg/day of fluticasone, FP) has been shown to reduce airway vasculature in asthma patients [261]. In particular, ICS decreased the vessel number by 30%, VEGF staining by 40%, and angiogenic sprouting by 25%.

TSLP is expressed in the airways of COPD patients [107], and it is overexpressed in ASM from COPD patients compared to healthy subjects [111]. TSLP induces the release of angiogenic and lymphangiogenic factors from HLMs [20]. Tezepelumab is a monoclonal antibody [87] anti-TSLP that has been shown to improve lung function in severe asthma [262,263]. The efficacy of tezepelumab in preventing COPD exacerbations is presently under investigation (NCT04039113).

IL-33 concentrations are increased in serum, sputum, and bronchial biopsies of COPD patients [115,116,117,118,119], and serum IL-33 levels are associated with COPD exacerbations [120]. Itepekimab, a mAb anti-IL-33, has been shown to improve lung function in severe asthma [264]. IL-33 can induce angiogenesis in different experimental models [122,123]. mAbs targeting IL-33 (NCT03546907, NCT04751487, NCT04701983, NCT03096795) or ST2 (NCT05037929, NCT03615040) are under investigation in COPD patients.

Different classes of inhibitors targeting specific angiogenic factors (e.g., VEGFs, ANGPTs) and their receptors (i.e., VEGFRs, Tie1/2) have been developed to inhibit angiogenesis and lymphangiogenesis [265,266]. These compounds, some of which have already been approved for clinical use, could be used to evaluate the role of angiogenesis/lymphangiogenesis in preclinical and clinical models of COPD.

## 10. Closing Thoughts

Although several studies have investigated the role of vascular remodeling in COPD and have added new insights into the pathogenesis of chronic inflammatory airway diseases, there are still several unanswered questions (Table 1).

**Figure 4:**
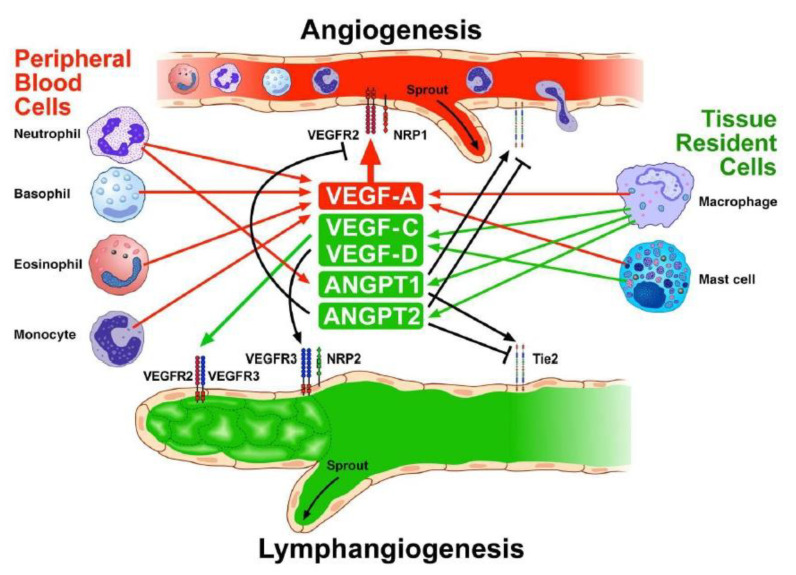
Schematic representation of the contribution of peripheral blood and tissue-resident immune cells in the modulation of angiogenesis and lymphangiogenesis in COPD. Macrophages, the predominant immune cells in human lung parenchyma, release angiogenic (VEGF-A) and lymphangiogenic factors (VEGF-C, -D) [20,45,137]. Human lung macrophages also release ANGPT1 and ANGPT2 [139]. Human lung mast cells release angiogenic (VEGF-A, -B) and lymphangiogenic factors (VEGF-C, -D) [9,144,145]. Human neutrophils contain and release several angiogenic factors, including VEGF-A and ANGPT1 [46,58,156]. Human basophils contain and release VEGF-A [157]. Human eosinophils synthesize and store in their granules VEGF-A [167]. Peripheral blood monocytes release VEGF-A. VEGF-A signals mainly through the activation of VEGFR2, highly expressed in endothelial cells. VEGF-C and -D activate VEGFR3 on lymphatic endothelial cells. ANGPT1 is a Tie2 agonist, whereas ANGPT2 antagonizes ANGPT1 and VEGF-A at the Tie2 and VEGFR2, respectively.

There are two major sources of angiogenic factors in the inflamed lung: immune and structural cells. Immune cells, such as monocyte/macrophages [20,45,58,137], neutrophils [46,156], mast cells [9,12,144,145], eosinophils [168], DCs [194,204], and basophils [157], produce several angiogenic and/or lymphangiogenic factors. Structural cells, such as ECs [35,42] and VSMCs [225], also produce angiogenic factors. A wide spectrum of chemical (adenosine, PGE_2_) [267] or immunologic stimuli [268], local hypoxia [269], and microbial factors [145] relevant in COPD are potent activators of angiogenic factor release (Figure 4).

Angiogenesis, a canonical feature of inflammation [11,12,57], is also altered in COPD [102,103,104,105,106,247,248,270]. Vascular abnormalities [271] and enhanced bronchial expression of angiogenic factors [105] have been associated with COPD development. It remains to be demonstrated whether these altered vascular responses might be involved in the pathogenesis of parenchymal and vascular remodeling in different stages (e.g., early and/or late) of the disease. VEGF and its receptors VEGFR1 and 2 may be involved in peripheral vascular and airway remodeling in an autocrine or paracrine manner. This system may also be associated with epithelial cell viability during airway wall remodeling in COPD.

The importance of lymphangiogenesis in COPD remains largely unknown. Two important lung-resident immune cells such as human macrophages [45,58,137] and mast cells [12,144,145] are major sources of two main lymphangiogenic factors (VEGF-C and –D). Intriguingly, recent evidence indicates that VEGF-C, differently from VEGF-D, can contribute to the resolution of inflammation [272]. The role of distinct (VEGF-C vs. VEGF-D) lymphangiogenic factors in different stages and phenotypes of COPD remains to be explored.

Asthma and COPD are airflow limitation diseases with similar clinical manifestations but different pathophysiologic mechanisms. The two inflammatory disorders can be successfully differentiated in the vast majority of cases [126]. Sometimes the presence of eosinophils in COPD patients might indicate co-existing ACOS [159,160,161]. Several studies have reported increased bronchial vascularity and the overexpression of angiogenic factors in asthma [273,274,275]. A marked increase in VEGF was seen in tissues and biological fluids from asthmatics, and the levels correlated with disease severity, but inversely with airway hyperresponsiveness [276,277,278]. By contrast, low VEGF levels have been noted in emphysema and VEGF blockade caused emphysema in murine models [241]. Thus, some authors have suggested that VEGF excess contributed to an asthma-like phenotype of COPD and VEGF deficiency to the development of pulmonary emphysema [279].

Several drugs and monoclonal antibodies that target the VEGF-VEGFR and the ANGPT-Tie pathways are in clinical practice and development for oncological and inflammatory applications [265,280]. We would like to speculate that further investigations should evaluate whether the correction of altered angiogenesis/lymphangiogenesis may prove beneficial in treating chronic inflammatory airway diseases. There is some evidence that cysteinyl leukotriene (CysLT) receptor antagonists can alter vascular permeability by reducing angiogenic factor expression in the airways [281]. Finally, agents that specifically inhibit various angiogenic factors (VEGFs, ANGPTs, etc.) and their receptors (VEGFRs, Tie1/2) controlling angiogenesis and lymphangiogenesis may offer novel strategies for treating microvascular changes in COPD.

## Figures and Tables

**Table 1:** Unanswered questions about angiogenesis and lymphangiogenesis in COPD.

Are angiogenesis and lymphangiogenesis secondary to chronic inflammation and/or reparative processes, or an important early step in COPD? What do other members of the VEGF family (e.g., VEGF-B, -C, -D) besides VEGF-A do in COPD? Is the hyperproduction of VEGF induced by local hypoxia an adaptive phenomenon, or does it have a pathogenic role in COPD? What are the main immunologic stimuli that induce the release of angiogenic and lymphangiogenic factors from resident lung immune cells (macrophages, mast cells, neutrophils) in COPD? Could human inflammatory cells, under appropriate circumstances, produce anti-angiogenic factors (e.g., VEGF-A165b)? What are the roles of pro- and anti-angiogenic chemokines synthesized by human inflammatory cells in COPD? What part do alarmins (TSLP, IL-33, IL-25) play in angiogenesis in COPD? Are other angiogenic networks (e.g., ANGPT/Tie receptors) involved in COPD? What is the importance of lymphangiogenesis in COPD? What stimuli drive lymphangiogenesis during inflammation? MicroRNAs (miRNAs) regulate gene expression and specific miRNAs that regulate endothelial cell functions and angiogenesis have been described. Are specific miRNAs involved in angiogenesis/lymphangiogenesis in COPD?

## Abbreviations

α1AT, α1 anti-trypsin; ACOS, asthma-COPD overlap syndrome; ANGPT, angiopoietin; ASMC, alveolar smooth muscle cell; BAL, bronchoalveolar lavage; BEC, bronchial epithelial cells; bFGF, basic fibroblast growth factor; Breg, regulatory B cell; CAM, chorioallantoic membrane; CG, cathepsin G; COPD, chronic obstructive pulmonary disease; CRP, C-reactive protein; CSE, cigarette smoke extract; CysLT, cysteinyl leukotriene; cysLTR, cysLT receptor; DC, dendritic cell; EC, endothelial cell; EGFR, epithelial growth factor receptor; EPC, endothelial precursor cells; HGF, hepatocyte growth factor; HLM, human lung macrophage; HLMC, human lung mast cell; ICS, inhaled glucocorticoids; IL, interleukin; ILC, innate lymphoid cell; LEC, lymphatic endothelial cell; lfTSLP, long form TSLP; LPS, lipopolysaccharide; LT, leukotriene; LTRA, leukotriene receptor antagonist; miR, microRNA; mAb, monoclonal antibody; MMP, matrix metalloproteinase; MPO, myeloperoxidase; NE, neutrophil elastase; NET, neutrophil extracellular trap; NGF, nerve growth factor; NK, natural killer cells; NRP, neuropilin; PAF, platelet activating factor; PBMC, peripheral blood mononuclear cell; PC, pericytes; PlGF, placental growth factor; PR3, proteinase 3; RNA, ribonucleic acid; ROS, reactive oxygen species; sfTSLP, short form TSLP; SMC, smooth muscle cell; sPLA_2_, secretory phospholipase A_2_; sVEGFR1, soluble VEGFR1; Th17, T helper 17; TSLP, thymic stromal lymphopoietin; TSLPR, thymic stromal lymphopoietin receptor; TSP1, thrombospondin-1;VEGF, vascular endothelial growth factor; VEGFR, vascular endothelial growth factor receptor; VPF, vascular permeability factor; VSMC, vascular smooth muscle cell.
